# Insights into *Porphyromonas somerae* in Bladder Cancer Patients: Urinary Detection by ddPCR

**DOI:** 10.3390/microorganisms12102049

**Published:** 2024-10-10

**Authors:** Filippo Russo, Speranza Esposito, Lorella Tripodi, Savio Domenico Pandolfo, Achille Aveta, Felice Amato, Carmela Nardelli, Ciro Imbimbo, Lucio Pastore, Giuseppe Castaldo

**Affiliations:** 1 Department of Molecular Medicine and Medical Biotechnologies, University of Naples Federico II, 80131 Naples, Italy; filippo.russo3@unina.it (F.R.); speranza.esposito@unina.it (S.E.); lorella.tripodi@unina.it (L.T.); felice.amato@unina.it (F.A.); lucio.pastore@unina.it (L.P.); giuseppe.castaldo@unina.it (G.C.); 2 CEINGE Biotecnologie Avanzate—Franco Salvatore S.C.A.R.L., 80131 Naples, Italy; pandolfosavio@gmail.com (S.D.P.); achille-aveta@hotmail.it (A.A.); 3 Department of Neurosciences, Reproductive Sciences and Odontostomatology, University of Naples Federico II, 80130 Naples, Italy; ciro.imbimbo@unina.it; 4 Department of Urology, University of L’Aquila, 67010 L’Aquila, Italy; 5 Department of Urology, Ospedale del Mare, ASL NA1 Centro, 80147 Naples, Italy

**Keywords:** urobiome, bladder cancer, *Porphyromonas somerae*, ddPCR, NGS

## Abstract

To date, the increased awareness of the impact of microbes on human health has promoted scientific interest in microbiome studies for diagnostic and therapeutic purposes, revealing correlations between specific taxa and cancer. In particular, numerous species of *Porphyromonas* have been associated with several types of tumors. Previously, we studied the urobiome using Next-Generation Sequencing (NGS), and found an increase in *Porphyromonas somerae* in first morning urine of subjects affected by bladder cancer (BCa). Here, we aimed to confirm the presence of *P. somerae* in BCa patients by using droplet digital Polymerase Chain Reaction (ddPCR), testing a cohort of 102 male subjects over 50 years. Our findings showed a significant increase in *P. somerae* in the urine of the BCa group within both ddPCR and NGS, and a correlation between the two methods was observed at a statistical level. Moreover, *P. somerae’s* identification with ddPCR confirmed a significant association between this bacterium and the presence of BCa, highlighting its potential role as a biomarker. This allows us to propose the ddPCR as a suitable method for first-stage BCa screening and follow-up.

## 1. Introduction

Next-generation sequencing (NGS) has revolutionized genomics by enabling the rapid and comprehensive analysis of DNA and RNA molecules, providing unparalleled capabilities for identifying new genetic biomarkers, and advancing our understanding of complex biological systems and disease mechanisms [1,2,3]. In this context, the increase in our awareness of microbial communities’ role in human health and disease has led to growing scientific interest in studying their composition in different body niches [4]. This has also allowed for the study of the microbiome for diagnostic or therapeutic purposes [5,6]. More and more studies focused on microbiome characterization to understand the complexity of the interplay between dysbiosis and pathologies’ development and progression, due to the production of specific metabolites, the alteration of the inflammatory state, and the variation in the immune system response [7,8,9]. In particular, several microbiome analyses conducted in the oncological field highlighted the strong correlation between specific taxa and cancer. Notably, several recent studies evidenced the link between species of Porphyromonas genus and different oncological conditions: *P. gingivalis* with orodigestive and pancreatic cancers [10,11,12], *P. asaccharolytica* with colorectal cancer, and *P. endodontalis* with gastric adenocarcinoma [13]. Moreover, *Porphyromonas somerae* has been observed to increase the expression of proinflammatory cytokines and chemokines by endometrial cells, suggesting its contribution to the onset and progression of endometrial cancer [14]. For the first time, we found an increased abundance of *P. somerae* in the first morning urine of bladder cancer (BCa) patients, analyzing the urobiome of male patients with BCa over 50 years using 16S rRNA gene sequencing with Next Generation Sequencing (NGS) technology [15]. For the specific detection and quantification of *P. somerae* in bladder cancer diagnosis, we decided to take advantage of droplet digital Polymerase Chain Reaction (ddPCR). Indeed, the ddPCR has a higher sensitivity and specificity, quantitative precision, cost-effectiveness, faster turnaround times, reduced contamination risk, and simpler data analysis than NGS [16]. These features make ddPCR a powerful tool in clinical diagnostics where the targeted, accurate, and timely detection of pathogens is crucial. Here, we aimed to detect *P. somerae* by ddPCR in first morning urine samples of BCa patients.

## 2. Materials and Methods

### 2.1. Study Design

In the present study, we recruited 102 male subjects aged over 50 years. In total, 37 out the 102 were BCa-affected patients; 24/102 non-oncological patients were used as healthy controls (HCs); and 41/102 oncological patients, who were all affected by prostate cancer, which is the most common tumor in males over 50 years old in Italy, were used as non-healthy controls (NHCs). Subjects with recent urinary tract infections or those who had been under treatment with antibiotics in the last month were excluded from the study. The general characteristics of the study population are reported in Supplementary Table S1. All patients were admitted between 2021 and 2024 and they provided informed consent to participate in the study, which was approved by the Ethical Committee of the University Federico II of Naples (n. 191/20) and conducted following the Helsinki Declaration.

### 2.2. Bacterial DNA Extraction and ddPCR Design

Bacterial DNA was extracted from samples of first morning clean catch urine (including the first stream), using a MagPurix^®^ Bacterial DNA Extraction Kit (Zinexts Life Science, New Taipei City, Taiwan) via an automated system and according to manufacturer’s instructions. The yield and quality of extracted DNA were evaluated using the Qubit dsDNA High Sensitivity assay kit (Invitrogen Co., Life Sciences, Waltham, MA, USA) and the TapeStation (Agilent Technologies, Santa Clara, CA, USA), respectively [15]. The ddPCR experiments were conducted as previously described [17,18]. According to the manufacturer’s protocol, bacterial DNA was assembled in a Digital Droplet PCR reaction with 2× ddPCR Supermix for EvaGreen (BioRad, Hercules, CA, USA), using a primer pair specific for *P. somerae* detection: forward primer 5′-TGCGTAGGTGGCTGATTAAG-3′ and reverse primer 5′-AGTTTACGGCGTGGACTACC-3′ [19]. To confirm the presence of bacterial DNA in each analyzed sample, the 16S rRNA subunit gene was amplified using the forward primer 5′-ACTCCTACGGGAGGCAGCAGT-3′ and reverse primer 5′-TATTACCGCGGCTGCTGGC-3′. The quantification of positive and negative droplets generated from ddPCR was performed using QuantaSoft™ software version 1.7.4.0917 (BioRad, Hercules, CA, USA).

### 2.3. ddPCR vs. NGS Data Comparison

In order to compare ddPCR bacterial quantification with urobiome data from our prior research, the actual abundance of *P. somerae* was exported from raw NGS records obtained through 16S rRNA gene sequencing (PRJNA981420 project on Sequence Read Archive) [15]. Specifically, we considered urobiome data from 66 individuals, including 25 BCa patients, 24 HC, and 17 NHC patients.

### 2.4. Statistical Analysis

The Z-score standardization was applied to both ddPCR and NGS records, highlighting 2 outliers that were removed. Thus, we considered 100 and 64 samples for each dataset in the further analyses. Comparative analyses were performed by using the Kruskal–Wallis test, Mann–Whitney test, and the Chi-squared (*χ*^2^) test. The Spearman’s rank coefficient and Levene test were, respectively, used to evaluate the correlation and the variances’ equality between samples analyzed by both ddPCR and NGS. All statistical analyses and the construction of graphs were performed using GraphPad Prism (version 9.5.1).

## 3. Results

### 3.1. P. somerae Detection: ddPCR vs. NGS

**Figure 1:**
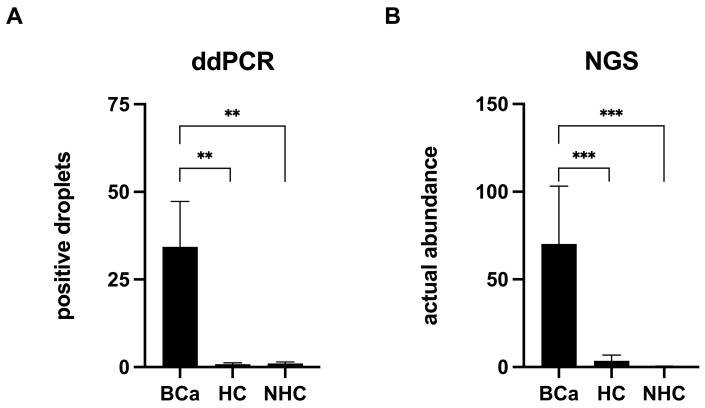
*Porphyromonas somerae* analysis with ddPCR and NGS. The mean of droplets positive for *P. somerae* identified with ddPCR (**A**) and actual abundance of *P. somerae* obtained by NGS analysis (**B**) were represented. Comparison analysis between the three groups (100 samples from ddPCR dataset: 35 BCa, 24 HC, 41 NHC; 64 samples from NGS dataset: 25 BCa, 24 HC, 17 NHC) revealed a statistically significant difference both for ddPCR (*p* = 0.003) and NGS (*p* < 0.0001). Pairwise comparisons confirmed significant differences between BCa and HC (ddPCR, *q* = 0.008; NGS, *q* < 0.0001) and BCa vs. NHC (ddPCR, *q* = 0.0014; NGS, *q* < 0.0001). BCa: bladder cancer group; HC: healthy control non-oncological group; NHC: non-healthy control group with prostate cancer. Data are shown as mean and standard error of mean. ** *q* < 0.01; *** *q* < 0.0001.

The presence of *P. somerae* evaluated using ddPCR showed a comparable result to that evaluated using NGS. A total of 100 samples were analyzed by ddPCR. The quantification of the bacterium was significantly increased in the BCa group (*n* = 35; mean count: 34.35), compared to the healthy (HC, *n* = 24; mean count: 0.85) and non-healthy (NHC, *n* = 41; mean count: 1.01) control groups (Kruskal–Wallis test, *p* = 0.003) (Mann–Whitney test; BCa vs. HC, *q* = 0.008; BCa vs. NHC, *q* = 0.0014) (Figure 1A). Data from 64 of these 100 samples were derived from the NGS analysis in our previous study. The actual abundance of *P. somerae* detected using NGS in the 25 BCa patients (mean actual abundance, mAA = 70.13) was significantly higher compared to the 24 HCs (mAA = 3.50) and 17 NHCs (mAA = 0.29) (*q* < 0.0001), following the same trend as the ddPCR analysis (Figure 1B). All 64 patients analyzed by NGS were also included in the ddPCR dataset, ensuring consistency between the two methods of analysis.

### 3.2. Correlation between ddPCR and NGS Methods in BCa Patients

A strong significant correlation between the number of positive droplets and the actual abundance of *P. somerae* was found in the BCa group when analyzing the samples tested with both methods (Spearman’s *r* = 0.78, confidential interval: 0.53 to 0.90; *p* = 0.0002) (Supplementary Figure S1). No significant difference in the variances between ddPCR and NGS was detected (Levene test, *p* > 0.05).

### 3.3. Association between P. somerae and Bladder Cancer

The results from 100 samples analyzed by ddPCR are reported in Table 1. In total, 29 out of the 100 patients tested positive for *P. somerae*, and 62.1% of them (18/29) were affected by BCa. Specifically, 51.4% of the BCa patients (18/35) had a positive result for the presence of the bacterium, dissimilar to both the HC and NHN groups in which the positivity resulted in only 20.8% (5/24) and 14.6% (6/41) of subjects, respectively. Nevertheless, a significant association was found between the presence of *P. somerae* and bladder cancer (*χ*^2^ = 13.44; *p* = 0.0012).

## 4. Discussion

Our study provides compelling evidence supporting the association between *P. somerae* and bladder cancer. Utilizing both ddPCR and NGS methodologies, we demonstrated that the presence and abundance of *P. somerae* in urine samples of BCa patients was significantly higher than in the urine samples from controls. This consistency across two different analytical techniques reinforced the reliability of our findings and underscored the potential of *P. somerae* as a biomarker for bladder cancer. To date, several studies have highlighted the link between specific *Porphyromonas* species (*P. gingivalis*, *P. asaccharolytica*, *P. endodontalis*, and *P. somerae*) and various types of cancer [10,11,12,13,14]. Specifically, *P. somerae* has shown the ability to colonize endometrial epithelium in patients with cancer, where protective microbial species, such as lactobacilli, were decreased [19]. Similarly, our previous research also highlighted an increased abundance of this bacterium in the urine microbiomes of patients in which protective species were underrepresented [15]. This suggests that *P. somerae* could opportunistically colonize and persist in environments with threated barriers, such as in cancer conditions. Furthermore, *P. somerae* seemed to contribute to the onset and progression of endometrial cancer, by increasing the expression of proinflammatory cytokines and chemokines in endometrial cells [14]. Crooks TA et al. demonstrated that *P. somerae* could invade epithelial cells in endometrial cancer models, evading the host immune system and promoting chronic inflammation through the production of succinate, a metabolic intermediate that stabilizes hypoxia-inducible factors, thereby contributing to cancer development via inflammation and angiogenesis [20]. In summary, the mechanisms through which *P. somerae* is associated with bladder cancer remain complex and not yet fully understood, and further studies are essential to clarify the possible mechanisms through which *P. somerae* is associated with bladder cancer.

## 5. Conclusions

Our findings contribute to reinforcing the association between *P. somerae* and bladder cancer. To date, ddPCR offers numerous advantages, including enhanced sensitivity and specificity, a high quantitative precision, cost-effectiveness, expedited turnaround times, a reduced risk of contamination, and simplified data analysis. Globally, these features make ddPCR a robust tool for targeted pathogen detection in clinical diagnostics. Indeed, our results showed that ddPCR matched the performance of NGS in detecting *P. somerae*, offering practical benefits for routine clinical application. We propose considering the use of ddPCR for rapid detection of *P. somerae* in the screening and follow-up of BCa. We are aware that the identification of *P. somerae* using NGS was carried out by sequencing a larger amplicon than that used for the ddPCR with the primers reported by Walsh et al. [19]. This could explain the lower detection of *P. somerae* in a specimen with a low biomass, such as urine. Further studies are needed to elucidate the pathogenic mechanism of *P. somerae* in bladder cancer.

## Figures and Tables

**Table 1:** *P. somerae* tested by ddPCR. For each of the three groups (BCa, HC, and NHC), the following results were reported: the number of samples that tested positive or negative for *P. somerae* by ddPCR (Count), and the percentage of positive and negative subjects compared to the total number of subjects with the same outcome (% within the same outcome).

		BCa	HC	NHC	Total (ddPCR Outcome)
**ddPCR Positive**	Count	18	5	6	**29**
	*% within positive outcome*	*62.1%*	*17.2%*	*20.7%*	
**ddPCR Negative**	Count	17	19	35	**71**
	*% within negative outcome*	*23.9%*	*26.8%*	*49.3%*	
	**Total** **(per group)**	**35**	**24**	**41**	**100**

BCa: bladder cancer group; HC: healthy control non-oncological group; NHC: non-healthy control group with prostate cancer.

## Data Availability

The original contributions presented in the study are included in the article, further inquiries can be directed to the corresponding author.

## Supplementary Materials

The following supporting information can be downloaded at: https://www.mdpi.com/article/10.3390/microorganisms12102049/s1, Figure S1: Correlation between ddPCR and NGS methods in BCa patients. The scatter plot represents the correlation of *P. somerae* quantification results obtained by ddPCR and NGS. A logarithmic scale was applied to both axes to better visualize the variability among the two methods, and an offset of 0.01 was added to all values in order to accommodate zero counts. The correlation was analyzed using Spearman’s rank correlation coefficient (Spearman’s *r* = 0.78, confidential interval: 0.53 to 0.90; *p* = 0.0002), showing a strong positive correlation between the two methods. ddPCR: droplet digital PCR; NGS: next-generation sequencing; Table S1: General characteristics of the study population.
