# Supplementary material for: Insights into Porphyromonas somerae in Bladder Cancer Patients: Urinary Detection by ddPCR
Source: Microorganisms. 2024 Oct 10;12(10):2049. doi: 10.3390/microorganisms12102049 (PMC11509927; doi:10.3390/microorganisms12102049)
Supplement: Supplementary file 1 [file microorganisms-12-02049-s001.zip › microorganisms-3218185-supplementary.pdf]

Supplementary Files

Supplementary Table S1. General characteristics of the study population.

Group		*Age* *(years ± SD)*	*BMI* *(Kg ± SD)*	*Tobacco* *(consumers)*	*Alcohol* *(consumers)*	*Diabetes* *(affected)*	*Hypertension* *(affected)*	**Tumor characteristics**			
**BCa**	**n=37**	73.1 ± 8.8	27.0 ± 3.0	11	8	11	17	**Bladder tumor**			
								TNM		*Grading*	
								Ta	14	Gx	1
								Tis	1	G1	10
								T1	16	G3	26
								T2	3		
								T2a	2		
								T2b	1		
**HC***	**n=24**	61.6 ± 7.6	-	-	-	-	-	**None**			
**NHC**	**n=41**	67.8 ± 6.2	25.1 ± 3.4	22	19	12	16	**Prostate tumor**			
								ISUP grade			
								Grade 1	28		
								Grade 22	12		

*For the HC group BMI, tobacco, alcohol, diabetes, and hypertension information were not reported because they were considered exclusion criteria.

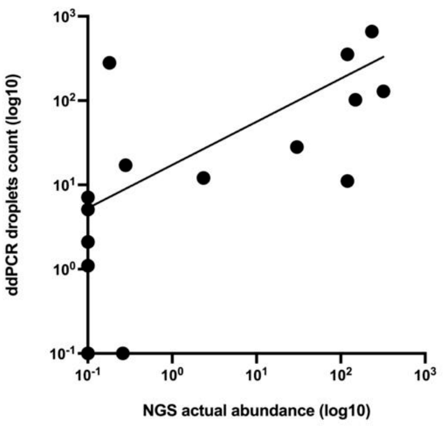

Supplementary Figure S1. Correlation between ddPCR and NGS methods in BCa patients. The scatter plot represents the correlation of *P. somerae* quantification results obtained by ddPCR and NGS. A logarithmic scale was applied to both axes to better visualize the variability among the two methods, and an offset of 0.01 was added to all values in order to accommodate zero counts. The correlation was analyzed using Spearman's rank correlation coefficient (Spearman's r = 0.78, confidential interval**:** 0.53 to 0.90; p = 0.0002), showing a strong positive correlation between the two methods. ddPCR: droplet digital PCR; NGS: next-generation sequencing.
